# Probing penile hemodynamics by using photoplethysmography as objective indicators for male erection quality and sexual function

**DOI:** 10.1038/s41598-021-91582-9

**Published:** 2021-06-08

**Authors:** Yuan-Hung Pong, Yi-Kai Chang, Ching-En Hsu, Po-Cheng Chen, Yu-Chuan Lu, Vincent F. S. Tsai, Hong-Chiang Chang, Men-Tzung Lo, Chen Lin

**Affiliations:** 1 Department of Biomedical Sciences and Engineering, National Central University, Taoyuan, 320 Taiwan; 2 Department of Urology, Ten Chen Hospital, Taoyuan, 320 Taiwan; 3 Department of Urology, National Taiwan University Hospital, Taipei, 100 Taiwan

**Keywords:** Urology, Urogenital diseases

## Abstract

Erectile dysfunction (ED) is mostly due to the lack of blood flow into the penis. In the past 20 years, near-infrared spectroscopy (NIRS) was used in measuring the concentrations and temporal dynamics of different hemoglobin types. However, the dynamics of the light absorption (photoplethysmography; PPG) have not been applied to survey penile hemodynamics and erection quality. This paper compared the use of photoplethysmography (PPG) to study vascular ED with standard penile Doppler ultrasonography. Men diagnosed with vascular ED for at least 6 months and nominated for penile ultrasonography were included. PPG signals were collected during the ultrasound examination. All beat-to-beat PPG waveforms were aligned with the peak and averaged to one representative *template waveform* for feature analysis, including amplitude differences (APD) index, reflection time index (RTI), augmentation index (AI), and perfusion index (PI). An inverse correlation was found between end-erection amplitude and both erection hardness score (EHS) and resistive index (RI). APD index and EHS as well as the international index of erectile function-5 (IIEF) and RI were positively correlated. RTI and AI were inversely correlated to IIEF and RI. PI was positively correlated to RI. PPG may therefore be useful as a noninvasive, convenient, technique for sexual function evaluation.

## Introduction

Erectile dysfunction (ED) is a condition that causes a negative impact on the quality of life, self-esteem, and partner relationship. It is defined as the inability to achieve and maintain an erection hard enough for intercourse to the mutual satisfaction of the man and his partner^1^. It influences a large population worldwide, with an estimated 320 million men affected with ED by 2025^2^. The failure of sustaining penile rigidity is mostly due to the lack of blood flow into the penis. Thus, penile hemodynamic measurements are fundamental to erectile function evaluation. Doppler ultrasonography and the pudendal vessel angiography are currently used to evaluate penile hemodynamics^3,4^. Most examination methods are invasive and inconvenient, making patients uncomfortable.

Near-infrared spectroscopy (NIRS), which uses light in the 700- to 1,000-nm wavelength, has been used to continuously assess changes in hemoglobin concentrations in the blood with very low energy^5^. In the last 20 years, NIRS has been used in urological examinations, including the measurement of testicular and renal cortex oxygenation, bladder functional studies, and penile hemodynamics^6–10^. In addition to measuring the concentrations of oxy- and deoxy-hemoglobin, the temporal dynamics of light absorption (photoplethysmography; PPG) is proportional to the volume changes in the arterial blood after removing the constant term due to the light absorption of tissue and the venous hemoglobin^11^. The dynamics of arterial light absorption measured in a finger were used as an alternative to noninvasive hemodynamic parameters in assessing vascular stiffness and continuous blood pressure in many studies^12,13^. PPG is ideal for in-hospital or home monitoring because most modern portable pulse oximetry equipment can export PPG signals. However, potential PPG applications in penile hemodynamics had not been studied. This study hypothesized that the reduction in the blood flow of cavernous arteries due to high pressure inside the penis during erection causes a relatively lesser blood volume pumped into the penis and induces a decrease in PPG amplitude, representing the degree of hardness of penile erection. Furthermore, the penile PPG waveform, an alternative to the pressure waveform of cavernous arteries, may also advance the current understanding of penile hardness, penile Doppler parameters, and sexual ability. This study was undertaken to investigate penile hemodynamics and penile erection quality using PPG.

## Results

### Patient demographics

Table 1 shows the demographics of the current study cohort. The mean age was 57.14 ± 13.05 years, and the mean international index of erectile function-5 (IIEF-5) score was 11.89 ± 4.52. Most subjects in this study presented an erection hardness score (EHS) score 2 and inability to complete intercourse in the past 6 months. Most of the ED cases were observed in the mild to moderate groups. Significant differences exist in the amplitude differences (APD) index, reflection time index (RTI), augmentation index (AI), and resistive index (RI) when patients were grouped by ED severity (Table 2). The moderate and severe ED groups had smaller perfusion index (PI) values and lower peak systolic velocities, although the latter was not statistically significant.

**Table 1:** Basic demographics of all patients.

Basic demographics of all patients	All patients (N = 68)
Age	57.14 ± 13.05
Height	167.79 ± 4.94
Weight	76.75 ± 11.43
BMI	27.28 ± 4.21
IIEF	11.89 ± 4.52
**EHS**	
1	9 (13.23%)
1.5	6 (8.82%)
2	20 (29.41%)
2.5	6 (8.82%)
3	6 (8.82%)
3.5	10 (14.70%)
4	11 (16.17%)
**Erectile dysfunction severity**	
Mild	9 (13.23%)
Mild to moderate	28 (41.17%)
Moderate	19 (27.94%)
Severe	12 (17.64%)
**Comorbidities**	
Hypertension	22 (32.35%)
Dyslipidemia	22 (32.35%)
Diabetes mellitus	21 (30.88%)
Prostate cancer	14 (20.58%)
Cardiac disease	6 (8.82%)
Hyperthyroidism	4 (5.88%)
Others	2 (2.94%)

*BMI* Body mass index, *IIEF* International index of erectile function, *EHS* Erection hardness score.

**Table 2:** The comparison of BMI, PSV, APD, and penile waveform series in ED severity.

Parameters	BMI	PSV	APD index	Reflection time ratio (RTI)	Augmentation index (AI)	Perfusion index (PI)	Resistive index (RI)
Mild ED (n = 9)	27.36 ± 4.36	43.90 ± 21.59	0.118 ± 1.579^d^	0.234 ± 0.102^c^	0.297 ± 0.200^c^	0.463 ± 0.655	0.903 ± 0.056
Mild to moderate ED (n = 28)	27.42 ± 4.24	43.64 ± 20.82	− 0.083 ± 1.734^d^	0.243 ± 0.098	0.319 ± 0.204	0.457 ± 0.641	0.886 ± 0.082^c,d^
Moderate ED (n = 19)	27.27 ± 4.21	43.71 ± 20.39	− 0.094 ± 1.730^d^	0.244 ± 0.098^a^	0.320 ± 0.210^a^	0.444 ± 0.306	0.883 ± 0.081^b^
Severe ED (n = 12)	27.18 ± 3.99	43.63 ± 21.72	− 0.149 ± 1.771^a,b,c^	0.240 ± 0.096	0.304 ± 0.193	0.345 ± 0.545	0.880 ± 0.080^b^
*ANOVA*			0.004	0.005	0.037	0.619	0.009

*BMI* Body mass index, *PSV* Peak systolic velocity of cavernous artery.

^a^Post-hoc test *p* < 0.05 compared with mild ED.

^b^Post-hoc test *p* < 0.05 compared with mild to moderate ED.

^c^Post-hoc test *p* < 0.05 compared with moderate ED.

^d^Post-hoc test *p* < 0.05 compared with severe ED.

### Amplitude correlative series and sexual function

**Figure 1:**
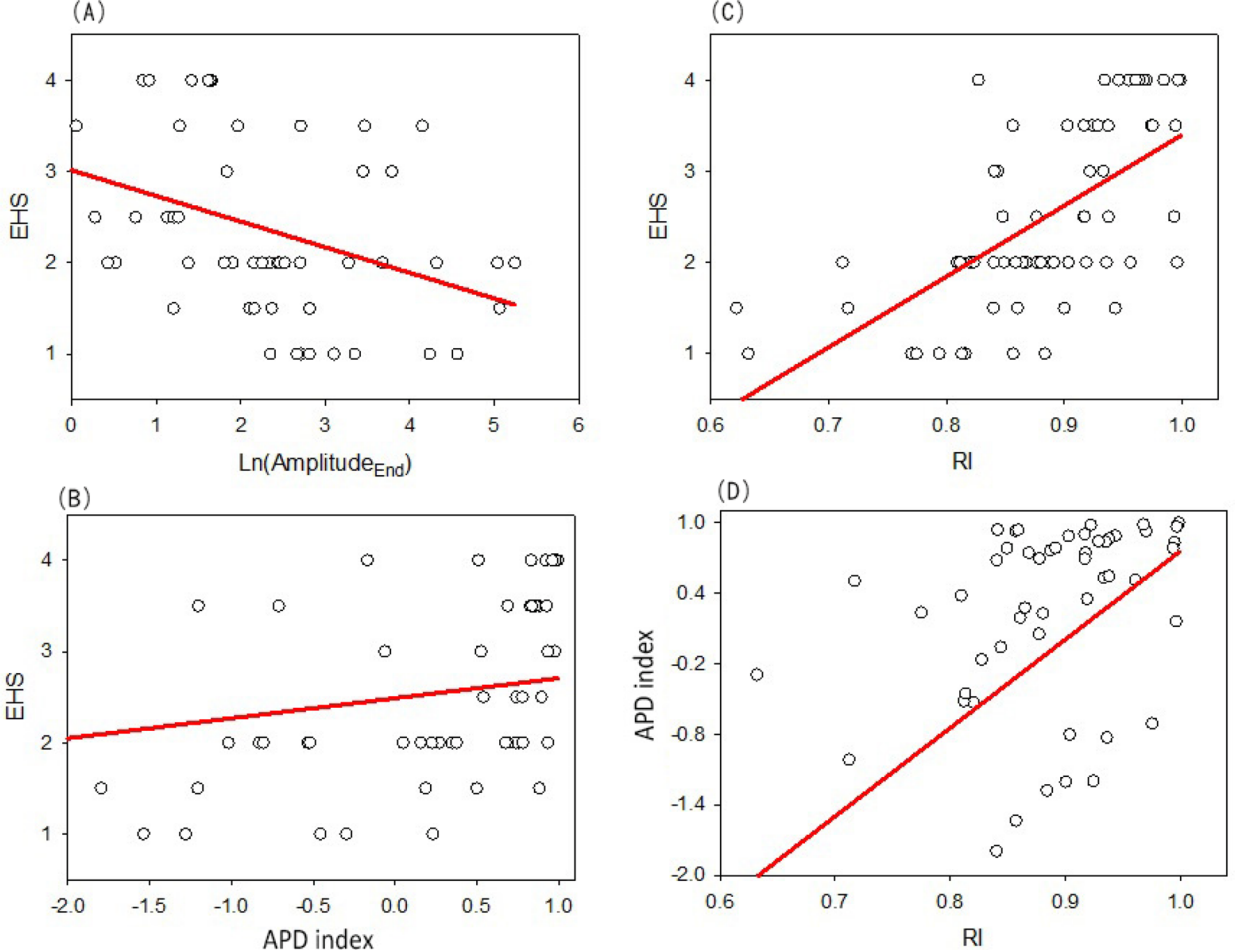
The amplitude correlative series and sexual function. A: Amplitude in the end-erection period is negatively correlated with EHS (*R* =  − 0.405, *p* < 0.05); B: APD index is positively correlated with EHS (*R* = 0.522, *p* < 0.05); C: RI is positively correlated with EHS (*R* = 0.670, *P* < 0.001); D: APD index is positively correlated with RI (*R* = 0.417, *p* < 0.05).

Penile erection was verified to correspond to decreased PPG signals. The amplitude in the end-erection period, as well as EHS and RI, had a significant inverse relationship (Table 3, Fig. 1). The relationship between RI and EHS in this study was also statistically significant (*R* = 0.670, *P* < 0.001). Conversely, the APD index is positively correlated with IIEF, EHS, and RI (Fig. 1, Table 3). A higher intracavernosal pressure (ICP) at the end of the erection, which represents the harder penis, causes less blood volume into the penis resulting in lower end-erection PPG amplitude and higher APD index (the difference between mid- and end-erection amplitudes). Nevertheless, the correlation between the PPG amplitude and peak systolic velocity of the cavernous artery was not statistically significant (*p* = 0.921).

**Table 3:** The correlation among PPG signals and clinically observed parameters.

R value	BMI	IIEF	EHS	PSV	EDV	RI
End-erection amplitude	0.135	− 0.151	− 0.405*	− 0.002	0.119	− 0.151^#^
APD index	− 0.114	0.289*	0.522*	− 0.149	− 0.553*	0.417*
RTI	0.198*	− 0.362*	− 0.181^#^	− 0.029	0.207	− 0.265*
AI	0.233*	− 0.305*	− 0.095	− 0.007	0.086	− 0.148
PI	0.023	0.026	0.136	− 0.077	− 0.437*	0.338*

*EDV* End-diastolic velocity in “end-erection” status.

*p < 0.05; ^#^*p* = 0.09.

### The waveform characteristics and sexual function

**Figure 2:**
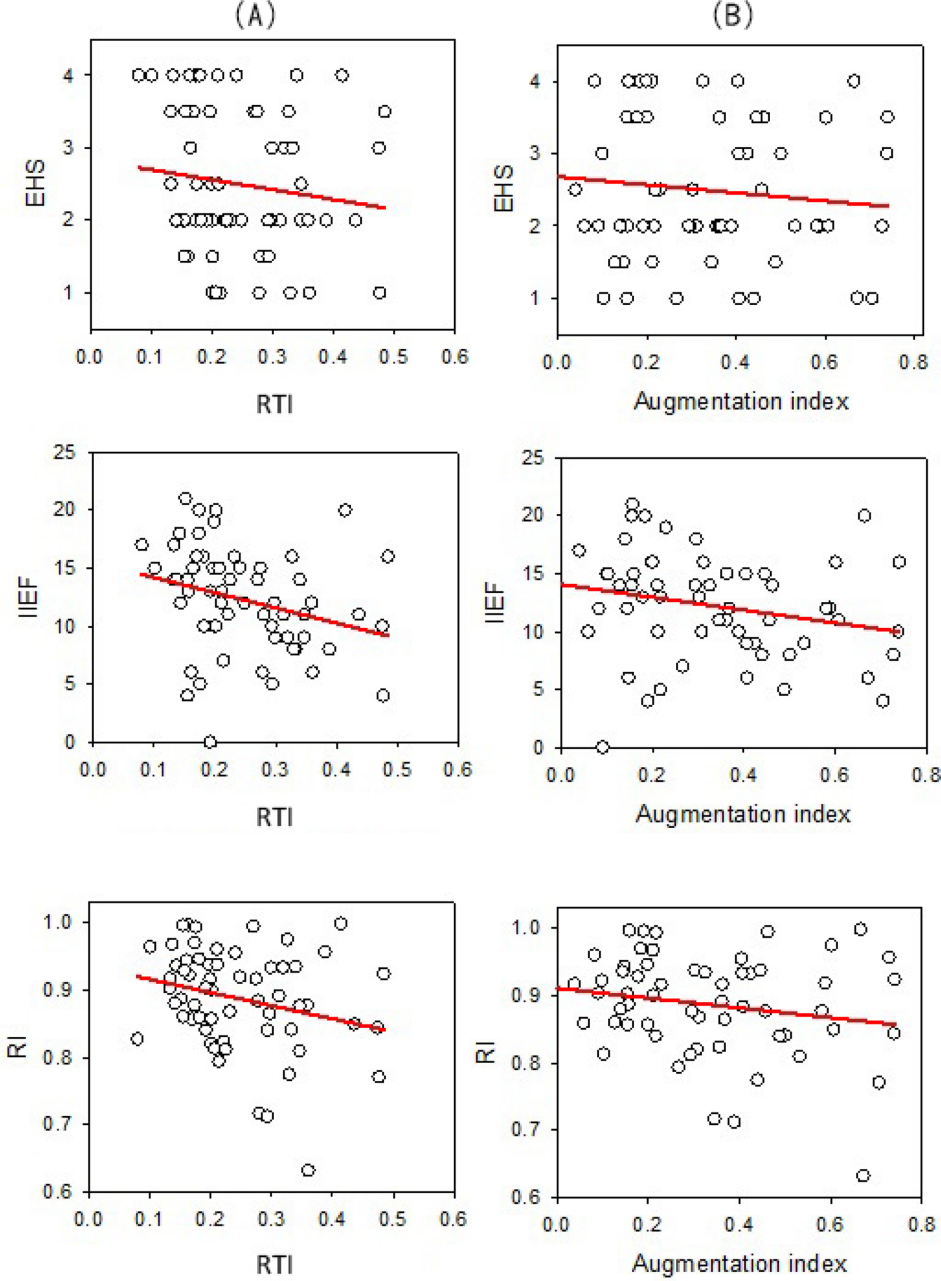
The waveform series and sexual function. A: Negative trends among RTI, EHS, IIEF, and RI; B: negative trends among AI, EHS, IIEF, and RI.

The cavernous arterial RTI and AI were negatively correlated with IIEF and RI (Table 3, Fig. 2). The PI was positively correlated with RI and inversely correlated with end-diastolic velocity. These findings support that the harder cavernous tissue (higher EHS or penile hardness) can stiffen the local cavernous artery and cause the PPG waveform changes. The main and the reflected wave are superimposed earlier, leading to a higher second peak and shorter reflection time resulting in lower AI and RTI, because the pulse wave travels faster in the stiffened artery. The perfusion index, considered as the dynamic acceleration of arterial vessel wall, was also observed to may be an indicator of RI and end-diastolic velocity.

## Discussion

This study analyzed the dynamics of arterial PPG monitored via NIRS and derived several waveform characteristics. First, the PPG amplitude in the end-erection period can be an indicator for EHS, and the APD index of the cavernous artery is positively correlated with IIEF, EHS, and RI. The APD index was significantly lower in the severe ED group compared with the other groups. Second, during the mid-erection period, the RTI and AI derived from the systolic and diastolic peaks of the waveform and the stiffness indexes for the vascular system were negatively correlated with IIEF and EHS. Third, the PI value, a measurement of the dynamics of vessel wall changes, is positively correlated with RI and inversely correlated to end-diastolic velocity. All these findings can be translated to a higher ICP built at the end of the erection leading to lesser blood volume into the penis and hardens it. These physiological changes during erection can be assessed by the amplitude parameters (low amplitude at the end-erection period and high APD index) and waveform parameters (lower AI and lower RTI) derived from PPG.

ED is a problem in obtaining or maintaining an erection for sexual intercourse. The prevalence of ED increases with age and is becoming continuously frequent in young males in recent years^14^. Aside from PPG, clinicians use several noninvasive devices to gather erectile information, including strain gage (e.g., Erektor), pulse–volume–plethysmography, and laser Doppler imaging^15–18^. PPG use for penile hemodynamics has several distinct advantages over the other aforementioned devices. First, PPG can potentially measure not only changes in hemoglobin in oxygenated blood^19^ but also arterial volume changes (proportional to light absorption dynamics). Second, it is low cost, portable for natural settings (e.g., in actual human intercourse context instead of video stimulations), which makes long-term recording possible in a clinical friendly environment, and is more comfortable for patients. Recent urological studies using NIRS have included testicular ischemia, voiding dysfunction, and ED^6,9,20–28^. Kim et al. performed a NIRS experiment with six healthy men shown an adult video for 4 min^28^. The NIRS sensors for collecting signals were placed on the side and top of the penis. A significant increase of oxygenated hemoglobin (HbO) when the adult video started indicated the dilation of the cavernous arteries and the compression of the penile venous complex. A similar NIRS study was undertaken by Kudlow et al. to compare hemoglobin concentrations in healthy young men and prostate cancer patients after bilateral nerve-sparing radical prostatectomy^27^. They found that HbO increased up to 70% and 58.3% in the young, healthy group and in the prostate cancer groups, respectively. These studies suggest that NIRS can be a convenient method that is sensitive enough for detecting real-time hemoglobin concentration changes in the dilated arterial system in various locations of the penis during visual sexual stimulation with real-time results. However, the proponents did not address the relationship between the dynamic changes in oxygenated hemoglobin concentrations and erectile function or penile arterial profiles. Consequently, a study on penile saturation during flaccid and erection periods in men with and without ED was conducted in 2007 by Padmanabhan et al., who found that men with ED have significantly lower corporal and glandular penile saturation during the flaccid period^26^. The study explored the relationship between resting saturation and erectile function but did not discuss data on the erection period. None of the above studies analyzed the dynamics of chromophores associated with arterial volume changes (Supplementary material Table S1).

EHS was negatively associated with the PPG amplitude in the end-erection period and positively correlated with RI. The correlation between EHS and RI can be attributed mostly to end-diastolic velocity during the end-erection period but not to maximal systolic velocity although a significant increase in maximal systolic velocity and a significant decrease in end-diastolic velocity were observed. Thus, penile hardness needs cumulative pressure in the engorged corporal tissue during end-erection. Burnett et al. established a series of spectral changes correlated to the volume change of the in vitro model system and use the optical signal changes (light absorption) to predict the actual penile blood volume change. They found that the blood volume percentage increase is correlated with erection quality (*P* < 0.001), penile rigidity (*P* < 0.005), and penile circumference increase (*P* < 0.0001). Additionally, the positive correlation between APD index and EHS indicates that penile hardness can be attributed to the dilation of the cavernous arteries during mid-erection and high ICP during the end-erection period during which engorged corporal tissue prevents blood to pass through or return to the venous system.

Peripheral PPG waveform (e.g., finger PPG) can be regarded as an alternative to arterial pressure waveform^12^. The decreased AI and RTI during the resting state can be a result of reduced compliance of the elastic arteries, thus causing an earlier return of the reflected wave^12,29,30^. The harder cavernous tissue (higher EHS or penile rigidity) can stiffen the local cavernous artery and cause the reflected wave to return sooner. The earlier superimposed reflected peak leads to a higher second peak, resulting in lower AI and shorter reflection time and, thus, a lower RTI. Therefore, the lower cavernous arterial RTI and AI were associated with lower IIEF and RI. However, the AI and RTI during the end-erection period could not be determined because the differences between the systolic and diastolic volumes were minimal. Therefore, AI and RTI derived during the mid-erection period may not be a good predictor for penile hardness during end erection.

It is believed that this is the first study to analyze the dynamics of PPG light absorption waveform characteristics correlated with erectile function, penile hardness, and penile Doppler ultrasound profiles. These findings demonstrate that PPG may be useful as a noninvasive, potential technique to evaluate penile erection and men’s health given the advantages (e.g., low cost, transportability, continuous data acquisition, and safety). The findings of this study demonstrate that PPG signal dynamics can potentially be applied to clinical penile hardness and erectile function assessments. For example, the clinical use of PPG signal dynamics can extend to collect the nighttime erection status and hardness, i.e., nocturnal penile tumescence (NPT) test. Thus, conducting an NPT test at home is feasible and more convenient. Moreover, the penile erection status during intercourse may also be objectively evaluated. In combination with a self-reported questionnaire, the clinicians can better understand and judge the quality of sexual life. The application of PPG device might potentially be a new idea of at-home erection quality evaluation. However, this study has some limitations. Firstly, a relatively small sample size (*n* = 68) was studied. Nevertheless, this is the PPG study with the biggest sample size to date. Secondly, some correlations of the derived parameters were weak. Thirdly, a control group of normal, healthy subjects was not used for comparison because of the risks of alprostadil administration. More comprehensive studies in the future are warranted. Furthermore, *split-new* EHS was used instead of EHS score because of the following reasons. First, the *split-new* EHS score is determined by two urologists at the end of the penile ultrasound examination. Thus, the *split-new* EHS score is more detailed and accurate than the EHS score reported by the patients. Second, erection hardness is found between the two integers from time to time (e.g., EHS 1 and 2, EHS 2 and 3, or EHS 3 and 4). Giving an integer score was difficult. Thus, a score of 0.5 (e.g., EHS 1.5 and EHS 2.5 or 3.5) was given. Third, more groupings allow the understanding of the relationship between PPG and erection quality in more detail. Nonetheless, the current findings on PPG use in ED studies add insights to further understand erectile dysfunction, penile arterial stiffness, and men’s health.

In conclusion, this is the first study to explore penile hemodynamics, chromophore dynamics, and men’s health via PPG. This study demonstrated an inverse correlation between PPG amplitude at the end of erection and EHS. Positive correlations were found between the APD index and EHS as well as IIEF and RI. RTI and AI are inversely correlated to erectile function (IIEF). From a practical point of view, PPG has the potential to be a promising, convenient, noninvasive, and safe to evaluate penile erection and men’s health.

## Methods

All methods were carried out following relevant guidelines and regulations.

### Experimental subjects and paradigm

Men experiencing ED (IIEF < 22) for at least 6 months and nominated for penile Doppler ultrasonography were registered in this study. Patients were excluded with previous penile trauma, history of hormone replacement therapy, post-penile prosthesis, venous leak-type ED, and neurological ED. From November 2019 to June 2020, 68 men were enrolled at the National Taiwan University Hospital. Penile Doppler ultrasonography parameters, PPG data, and retrospective historical chart reviews were collected and approved by the National Taiwan University Hospital institutional review board (201907031RINA). All data were collected by two urologists with signed informed consent obtained from all participants in the study. Demographic data included age, height, body weight, BMI, endothelial risk factors (diabetes, hypertension, dyslipidemia, or medications), and personal medical and sexual histories.

### Experimental setup

**Figure 3:**
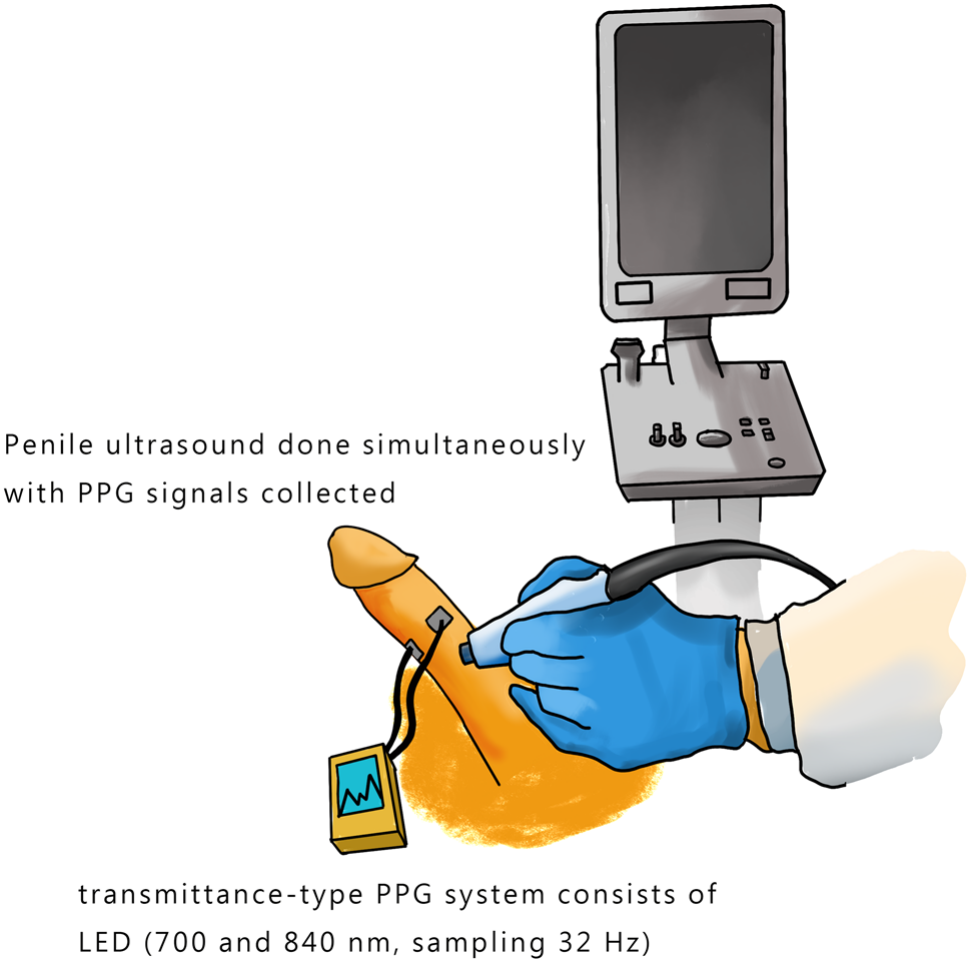
The experimental setup: The patients simultaneously receive penile Doppler ultrasound and transmittance-type PPG examination (700 and 840 nm, sampling 32 Hz) in the supine position. Penile Doppler ultrasonography is performed using high-frequency linear probes. The light-emitting diode (LED) and photodetector are included each in a separate sensor. They are both fixed to the penile root by stickers, leaving enough space for penile inflation. The LED and photodetector sensors are located on the skin of the front and back of the cavernous artery. The correct placement was guided by ultrasonography and confirmed by urologists.

Patients were laid down in the supine position, and 20 μg alprostadil (Caverject) was given via intracavernosal injection. PPG and penile Doppler ultrasonography were conducted 3 min after injection as *mid-erection* period signals. The PPG signals were collected for 2 min. PPG and penile Doppler ultrasonography were performed again for the *end-erection* period signals, also 2 min, when patients reached full-rigid penis status (Fig. 3). Penile Doppler ultrasonography was performed using high-frequency linear probes with the patient in the supine position. The peak systolic velocity (PSV) of the cavernous artery indicated that the highest speed detected at end-erection status (usually 20 min postpharmacostimulation) and the end-diastolic velocity (EDV) was also detected at the same time. RI is defined as the difference in PSV and EDV divided by PSV. RI helps predict venous leakage ED, which is excluded in the current population (RI, < 0.75). After completing the examinations, two urologists determined the *split-new* EHS right at the end of the penile Doppler test (*split-new* EHS scoring: 1, penis is larger but not hard; 1.5, the hardness score is between 1 and 2 or the penis tip score is 1 but the penis root score is 2; 2: penis is hard but not hard enough for penetration; 2.5: the hardness score is between 2 and 3 or the penis tip score is 2 but the penis root score is 3; 3: penis is hard enough for penetration but not completely hard; 3.5: the hardness score is between 3 and 4 or the penis tip score is 3 but the penis root score is 4; and 4: penis is completely hard and fully rigid).

### The PPG system and PPG waveforms

The light-emitting diode (LED) and photodetector are included each in a separate sensor. They are both fixed to the penile root by stickers, leaving enough space for penile inflation. The source detector distance is approximately 1.7 cm and increases with the inflation of the penis to 2.7 cm. The LED and photodetector sensors are located on the skin of the front and back of the cavernous artery. The correct placement was guided by ultrasonography and confirmed by urologists. The PPG system was approved by the Taiwan Food and Drug Administration and provided local tissue oxygen saturation and light absorption measurements sampled at 32 Hz. The transmittance-type PPG system consists of two LEDs at 700 nm and 840 nm as source of light and a light-dependent resistor as a photodetector. The PPG includes an amplifier, filter, and analog-to-digital converter. The light energy is absorbed by chromophores, i.e. mainly the oxy- and deoxy-hemoglobin in the corpus cavernosum when the light passes through the penile tissue. Thus, changes in the chromophore concentrations associated with hemodynamics were measured and recorded in real-time after removing the constant term (constant light absorption by the tissue and the venous hemoglobin, which is not pulsating), extracting a pulsatile PPG waveform. The instrument measures the arterial pulse wave, which is based on a higher chromophore concentration during systole and a lower concentration during diastole, which leads to a lower light intensity at the detector during systole and a higher one during diastole. The artery is dilated when blood pressure is increased. Thus, the real-time light absorption detected by the PPG detectors is correlated with the penile arterial pulsation. A second-order Butterworth band-pass filter was applied with a passband from 0.5 to 6.0 Hz to remove the noise.

### PPG signal preprocessing and features extraction

**Figure 4:**
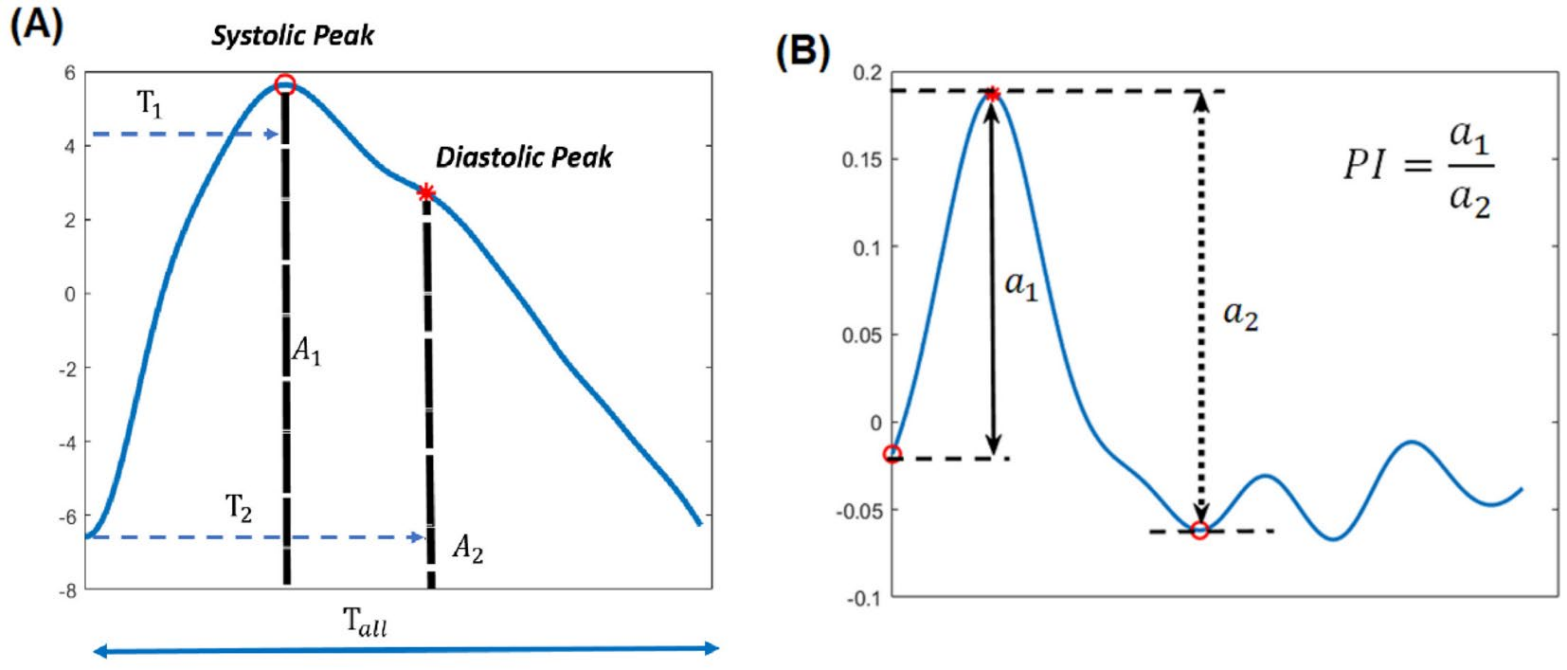
The calculation formula of reflection time index (RTI), augmentation index (AI), and perfusion index (PI). A: Template PPG waveform interpretation, *A1* amplitude of systolic peak, *A2* amplitude of diastolic peak, *T1* time to reach the systolic peak, and *T2* time to reach the diastolic peak B: Second derivative waveform transformed by the first-order differential of the template waveform for calculating PI.

The noise using a second-order Butterworth filter at 0.5–6.0 Hz and detected beat-to-beat systolic pulsatile waveforms was suppressed after decoding the PPG signals. All beat-to-beat PPG waveforms were aligned with the peak and averaged to one representative *template waveform* for further feature analysis (Fig. 4a). The template waveform was used to calculate the parameters of the chromophore dynamics index. A first-order differential of the *template waveform* was made as a *second derivative waveform* to evaluate the vessel wall changes acceleration (Fig. 4b).

The amplitudes (A1) of PPG signals (Fig. 4a) were calculated using template waveforms during the mid- and end-erection periods. APD is defined as the amplitude difference between the mid- and end-erection periods. The APD index is defined as the amplitude difference of the mid- and end-erection periods divided by the amplitude of the mid-erection period:

1 $$ {\text{APD}}\;{\text{index}} = \frac{{{\text{Amplitude}}\left( {{\text{mid}}} \right){-}{\text{Amplitude}}\left( {{\text{end}}} \right)}}{{{\text{Amplitude}}\left( {{\text{mid}}} \right)}}. $$

The fiducial points of the waveform are a consequence of a systolic pressure wave circulated forward to the peripheral vascular system being measured. In addition to the systolic pressure wave generated by the left ventricular contraction, the waveform also contains the second peak related to the reflected waves when the pressure wave reaches areas of turbulence or changes in lumen diameter (Fig. 4a)^15–17^. The RTI and AI of the arterial wave are widely used to assess vessel stiffness^31,32^. The RTI is defined as the time difference between the systolic and diastolic peaks in the arterial waveform (mid-erection period) divided by the total wave duration (Fig. 4a):

2 $$ {\text{RTI}} = \frac{{{\text{Diastolic}}\;{\text{peak}}\;{\text{time}} \left( {{\text{T}}2} \right) {-}{\text{ Systolic}}\;{\text{peak}}\;{\text{time}}\left( {{\text{T}}1} \right)}}{{{\text{Total}}\;{\text{wave}}\;{\text{duration}} \left( {{\text{tall}}} \right)}}. $$

The AI is defined as the amplitude difference between the systolic and diastolic peaks divided by the systolic peak in the mid-erection period (Fig. 4a):

3 $$ {\text{AI}} = \frac{{{\text{Systolic}}\;{\text{peak}}\;{\text{amplitude}}\left( {{\text{A}}1} \right){-}{\text{Diastolic}}\;{\text{peak}}\;{\text{amplitude}}\left( {{\text{A}}2} \right)}}{{{\text{Systolic}}\;{\text{peak}}\;{\text{amplitude}}\left( {{\text{A}}1} \right)}}. $$

Otherwise, the first-order differential of the template waveform was considered as a second derivative waveform to evaluate the changes in vessel wall velocity. PI is defined as the amplitude above the baseline in the second derivative waveform divided by the amplitude of the total wave (Fig. 4b):

4 $$ {\text{PI}} = \frac{{{\text{Amplitude}}\;{\text{above}}\;{\text{baseline}}\left( {{\text{a}}1} \right)}}{{{\text{Total}}\;{\text{amplitude}}\left( {{\text{a}}2} \right)}}. $$

### Statistical analysis

The baseline demographic characteristics are shown as the mean ± standard deviation for continuous variables and as proportions for categorical variables. Normality assumption was performed using the Shapiro–Wilk test in all the statistical tests. If the data present a normal distribution, the differences between groups were examined by one-way analysis of variance (ANOVA) with Tukey’s honestly significant difference post hoc comparison and Pearson’s correlation. Otherwise, the Kruskal–Wallis one-way ANOVA with pairwise post hoc comparison and Spearman’s rank correlation was applied for each of the measurements. All statistical analyses were performed using R software (version 3.5.0; R Foundation for Statistical Computing, Vienna, Austria) and IBM SPSS Statistics for Windows (version 22; IBM Corp., Armonk, NY, USA). A *p* value < 0.05 was considered statistically significant.

## Supplementary Information

Supplementary Information.
