# Supplementary material for: Probing penile hemodynamics by using photoplethysmography as objective indicators for male erection quality and sexual function
Source: Sci Rep. 2021 Jun 8;11:12019. doi: 10.1038/s41598-021-91582-9 (PMC8187730; doi:10.1038/s41598-021-91582-9)
Supplement: Supplementary file 1 — Supplementary Information. [file 41598_2021_91582_MOESM1_ESM.docx]

Table S1: Recent studies about erectile dysfunction (ED) and near-infrared spectroscopy (NIRS)

| Author and publication years | study subjects | nirs applications |
| --- | --- | --- |
| Burnett et al. (6) | 38 patients with ED | Diagnosis of vasculogenic erectile dysfunction |
| padmanabhan et al. (24) | 171 patients (ED: 144, non-ED: 27) | Men with ED have significantly lower corporal penile StO2. This may help further elucidate the relationship between corporal hypoxia and the development and progression of ED and possibly its treatment and prevention. |
| Kudlow et al. (25) | 29 Patients (ED: 12. Non-ED: 10) | There was a rapid rise in O2Hb concentration in men with excellent erections. In this preliminary study, we demonstrated that NIRS can be used to assess penile blood flow with sexual stimulation. In addition, more studies are needed to correlate NIRS testing with Doppler studies. |
| Kim et al. (26) | 6 healthy subjects | The outcomes demonstrate an ability of NIRS to be sensitive enough to detect the different  hemodynamic changes in various locations of a healthy male genital organ during visual sexual stimulation. The results also show the importance of sensor location on the genital organ for the resulting hemodynamic changes. |
